# Supplementary material for: The effects of island forest restoration on open habitat specialists: the endangered weevil Hadramphus spinipennis Broun and its host-plant Aciphylla dieffenbachii Kirk
Source: PeerJ. 2015 Feb 5;3:e749. doi: 10.7717/peerj.749 (PMC4327253; doi:10.7717/peerj.749)
Supplement: Appendix S1 [file peerj-03-749-s001.pdf]

Supplementary Materials

ITS2 primer design

For a subset of individuals, ITS2 was amplified using the primers ITS3 and ITS4 (646 to 660 base pairs) (White et al. 1990). The PCR cycle was 94°C for 3 min followed by 25 cycles of 94°C for 30 s, 60°C for 30 s and 72°C for 45 s, with a final extension at 72°C for 10 min. PCR products and a 1kb ladder were visualized on a 1.5% agarose gel stained with SYBR Safe™ DNA Gel Stain (Molecular Probes, Eugene, Oregon). For ITS2 there was non-specific low-molecular weight amplification and therefore the ~670 base pair band was gel purified (Ultrasep, Omega) prior to sequencing. PCR products were sequenced in both directions using 0.8 μM of primer, Big Dye version 3.1 (Applied Biosystems, Warrington, Cheshire, UK) and the following thermal regime: 96°C for 1 min followed by 25 cycles of 96 °C for 10 s, 50 °C for 5 s and 60°C for 4 minutes. Sequence products were resolved in an AVANT 3100 (ABI) capillary sequencer. The results were BLAST (Johnson et al. 2008) searched to confirm that they were ITS2 sequences.

References

Johnson, M., I. Zaretskaya, Y. Raytselis, Y. Merezhuk, S. McGinnis, and T. Madden. 2008. NCBI BLAST: a better web interface. Nucleic Acids Research **36**:5-9.

White, T. J., T. Bruns, S. Lee, and J. Taylor. 1990. Amplification and direct sequencing of fungal ribosomal RNA genes for phylogenetics. Pages 315-322 in M. A. Innis, D. H. Gelfand, J. J. Sninsky, and T. J. White, editors. PCR Protocols: A Guide to Methods and Applications. Academic Press, San Diego.
