# Supplementary material for: The effects of island forest restoration on open habitat specialists: the endangered weevil Hadramphus spinipennis Broun and its host-plant Aciphylla dieffenbachii Kirk
Source: PeerJ. 2015 Feb 5;3:e749. doi: 10.7717/peerj.749 (PMC4327253; doi:10.7717/peerj.749)
Supplement: Table S1 — GPS coordinates in degrees, decimal minutes of the Aciphylla dieffenbachii subpopulations surveyed on Rangatira and Mangere Islands. [file peerj-03-749-s003.docx]

|  | Subpopulation (Figures 2 and 3) | Coordinates (Degrees, Decimal Minutes) | |
| --- | --- | --- | --- |
|  |  | Southing | Westing |
| Rangatira | A | 44 21.288 | 176 10.009 |
|  | B | 44 21.189 | 176 10.425 |
|  | C | 44 20.232 | 176 10.758 |
| Mangere | 1 | 44 16.691 | 176 18.348 |
|  | 2 | 44 16.516 | 176 18.101 |
|  | 3 | 44 16.499 | 176 18.101 |
|  | 4 | 44 16.349 | 176 17.690 |
|  | 5 | 44 16.125 | 176 17.321 |
|  | 6 | 44 15.973 | 176 17.911 |
|  | 7 | 44 16.268 | 176 17.754 |
