# Supplementary material for: The effects of island forest restoration on open habitat specialists: the endangered weevil Hadramphus spinipennis Broun and its host-plant Aciphylla dieffenbachii Kirk
Source: PeerJ. 2015 Feb 5;3:e749. doi: 10.7717/peerj.749 (PMC4327253; doi:10.7717/peerj.749)
Supplement: Table S2 — GenBank accession numbers [file peerj-03-749-s004.docx]

|  | COI | CytB | ITS2 |
| --- | --- | --- | --- |
| A | KP664116 | KP664157 | KP664132 |
| B | KP664118 | KP664159 | KP664133 |
| C | - | - | KP664134 |
| D | KP664120 | KP664161 | KP664135 |
| E | - | - | KP664136 |
| F | - | - | KP664137 |
| G | KP664122 | KP664163 | KP664138 |
| H | KP664124 | KP664165 | KP664139 |
| I | - | - | KP664140 |
| J | KP664126 | KP664167 | KP664141 |
| M | - | - | KP664142 |
| N | KP664128 | KP664169 | KP664143 |
| O | KP664130 | KP664171 | KP664144 |
| 1.1 | - | - | KP664145 |
| 1.2 | KP664117 | KP664158 | KP664146 |
| 1.3 | - | - | KP664147 |
| 1.4 | KP664119 | KP664160 | KP664148 |
| 2.1 | - | - | KP664149 |
| 2.2 | KP664121 | KP664162 | KP664150 |
| 3.1 | KP664123 | KP664164 | KP664151 |
| 4.1 | KP664125 | KP664166 | - |
| 4.2 | - | - | KP664152 |
| 4.3 | KP664127 | KP664168 | KP664153 |
| 6.1 | KP664129 | KP664170 | KP664154 |
| 6.2 | KP664131 | KP664172 | KP664155 |
| 6.3 | - | - | KP664156 |

Table S2. GenBank accession numbers for COI, Cytb, and ITS2 genes.
